# Complete mitochondrial genomes of three Neotropical sleeper gobies: *Eleotris amblyopsis*, *E. picta* and *Hemieleotris latifasciata* (Gobiiformes: Eleotridae)

**DOI:** 10.1080/23802359.2017.1390412

**Published:** 2017-10-17

**Authors:** Fernando Alda, Alexandria J. Adams, W. Owen McMillan, Prosanta Chakrabarty

**Affiliations:** a Museum of Natural Science, Department of Biological Sciences, Louisiana State University, Baton Rouge, LA, USA;; b Smithsonian Tropical Research Institute, Panama, Republic of Panama

**Keywords:** Eleotrids, Middle America, mitogenome, Panama

## Abstract

We report the first complete mitochondrial genomes of three species of eleotrid fishes from the Pacific and Atlantic watersheds of Panama: *Eleotris amblyopsis*, *E. picta*, and *Hemieleotris latifasciata*. The three species have similar mitochondrial genomes with identical gene order; however, there are differences in the length of control region, 16S rRNA, and in seven of the tRNAs. In addition, ATP8 is one codon shorter in *E. picta* than in *E. amblyopsis* or *H. latifasciata*. We infer a phylogeny for Gobiiformes based on all mitochondrial protein-coding genes, which supports the monophyly of Eleotridae but does not recover Neotropical members of *Eleotris* as a distinct clade.

## Introduction

The freshwater fish fauna of Middle America contrasts to that of the Nearctic and South American regions in having a large number of salt tolerant species and marine taxa that have invaded the mainland, and are more or less permanent residents of freshwater streams (Myers 1966; Matamoros et al. 2015). This marine-derived and diadromous component comprises five families (Ariidae, Atherinidae, Gerridae, Eleotridae, and Gobiidae) that, despite representing an important fraction of the diversity of this Neotropical assemblage, is considerably understudied (McMahan et al. 2013; Galván-Quesada et al. 2016); particularly relative to the other taxa in the region such as poeciliids and cichlids that have been the focus of many systematic and biogeographic studies (e.g. Martin and Bermingham 1998; Perdices et al. 2005; Alda et al. 2013; Marchio and Piller 2013; McMahan et al. 2017).

In this study, we present the first complete mitochondrial genomes of three fish species of the family Eleotridae that are distributed across Atlantic and Pacific watersheds primarily in the Neotropics (but can be found as far north as California and South Carolina). In addition, we present a mitogenomic phylogeny of the Gobiiformes, which illustrates the position of the Neotropical members of the eleotrids in relation to other species for which complete mitochondrial genomes are available.

## Materials and methods

As part of ongoing studies on the systematics and biogeography of Neotropical freshwater fishes, we generated full mitogenomes of four individuals from three species of eleotrid fishes: *Eleotris amblyopsis* (STRI-16837, Quebrada Jobito, Río Indio, Coclé, Panama), *E. picta* (STRI-17754, Río Pichende, Río Piña, Darién, Panama; STRI-6991, Río Cate, Veraguas, Panama), and *Hemieleotris latifasciata* (STRI-7098, Río Tebario, Veraguas, Panama). Specimens were collected by electrofishing or seining, and gill arches were excised and preserved in a saturated 20% dimethyl sulphoxide (DMSO) and 0.5 M EDTA pH 8 solution at 4 °C. The whole specimens were fixed in formalin, vouchered and deposited in the Neotropical Fish Collection (NFC-STRI) at the Smithsonian Tropical Research Institute in Panama (STRI).

Mitogenome sequences were obtained as a by-product of a hybrid target capture of ultraconserved elements (UCEs) (Faircloth et al. 2012). We trimmed our reads for low-quality bases and adapter contamination using the parallel wrapper around Trimmomatic (Bolger et al. 2014) in illumiprocessor (Faircloth 2013). Then, we mapped our reads to reference mitochondrial genomes from the evolutionary closest species available using Bowtie 2 (Langmead and Salzberg 2012) implemented in Geneious 10.1.3 (www.geneious.com; Kearse et al. 2012). Using Geneious 10.1.3, we then created consensus sequences and annotated them for each sample.

We aligned our mitogenomes with representatives from five of the seven families of Gobiiformes (sensu Agorreta et al. 2013), and extracted all 13 protein-coding genes for subsequent phylogenetic analysis. We partitioned the data by gene and by codon, and estimated the best arrangement of partitions under a GTR + G nucleotide substitution model using PartitionFinder (Lanfear et al. 2012). We used these partitions in a Maximum Likelihood analysis in RAxML (Stamatakis 2014), that we run for 40 searches to find the best tree, and performed 500 bootstrap replicates to assess nodal support.

## Results and discussion

We obtained complete mitochondrial genome sequences from three representatives of the Eleotridae found primarily in the Neotropics: *E. amblyopsis*, *E. picta*, and *H. latifasciata*. All species genomes consist of 22 tRNA genes, two rRNA genes, 13 protein-coding genes, and control region (D-loop), arranged in identical order; their genome lengths are 16,519 bp, 16,547 bp, and 16,523 bp, respectively (Table 1). Full sequences are deposited in GenBank under accession numbers: MF927490 (*E. amblyopsis* STRI-16837), MF927491 (*E. picta* STRI-6991), MF927492 (*E. picta* STRI-17754), and MF927495 (*H. latifasciata* STRI-7098).

**Table 1.** Characteristics of the mitochondrial genomes of *Eleotris amblyopsis*, *E. picta*, and *Hemieleotris latifasciata*.

Code	Amino acid/gene	Start	Stop	Size	Spacer (+) or overlap (–)	Direction	Start codon	Stop codon
F	tRNA-Phe	1/1/1	68/68/68	68/68/68	0/0/0	F		
	12S rRNA	69/69/69	1021/1021/1021	953/953/953	0/0/0	F		
V	tRNA-Val	1022/1022/1022	1093/1093/1092	72/72/71	0/0/0	F		
	16S rRNA	1094/1094/1093	2780/2788/2782	1687/1695/1690	–1/–1/–1	F		
L	tRNA-Leu	2780/2788/2782	2853/2861/2856	74/74/75	0/0/0	F		
	ND1	2854/2862/2857	3828/3836/3831	975/975/975	+5/+4/+4	F	ATG/ATG/ATG	TAG/TAG/TAA
I	tRNA-Ile	3834/3841/3836	3903/3910/3905	70/70/70	–1/–1/–1	F		
Q	tRNA-Gln	3973/3980/3975	3903/3910/3905	71/71/71	–1/–1/–1	R		
M	tRNA-Met	3973/3980/3975	4042/4049/4043	70/70/69	0/0/0	F		
	ND2	4043/4050/4044	5089/5096/5090	1047/1047/1047	+1/+1/+1	F	ATG/ATG/ATG	TAA/TAA/TAA
W	tRNA-Trp	5091/5098/5091	5163/5169/5162	73/72/72	+2/+1/+2	F		
A	tRNA-Ala	5234/5239/5233	5166/5171/5165	69/69/69	+1/+1/+1	R		
N	tRNA-Asn	5308/5313/5307	5236/5241/5235	73/73/72	+36/+37/+37	R		
C	tRNA-Cys	5410/5416/5410	5345/5351/5345	66/66/66	0/0/0	R		
Y	tRNA-Tyr	5480/5487/5481	5411/5417/5411	70/71/71	+1/+1/+1	R		
	COXI	5482/5489/5483	7035/7042/7036	1554/1554/1554	0/0/0	F	GTG/GTG/GTG	TAA/TAA/TAA
S	tRNA-Ser	7106/7113/7107	7036/7043/7037	71/71/71	+3/+3/+3	R		
D	tRNA-Asp	7110/7117/7111	7181/7189/7182	72/73/72	+6/+5/+6	F		
	COXII	7188/7195/7189	7878/7885/7879	691/691/691	0/0/0	F	ATG/ATG/ATG	T–/T–/T–
K	tRNA-Lys	7879/7886/7880	7953/7960/7954	75/75/75	+1/+1/+1	F		
	ATP8	7955/7962/7956	8122/8126/8123	168/165/168	–10/–7/–10	F	ATG/ATG/ATG	TAA/TAA/TAA
	ATP6	8113/8120/8114	8795/8802/8796	683/683/683	0/0/0	F	ATG/ATG/ATG	TA-/TA-/TA-
	COXIII	8796/8803/8797	9580/9587/9581	785/785/785	0/0/0	F	ATG/ATG/ATG	TA-/TA-/TA-
G	tRNA-Gly	9581/9588/9582	9652/9659/9653	72/72/72	0/0/0	F		
	ND3	9653/9660/9654	10001/10008/10002	349/349/349	0/0/0	F	ATG/ATG/ATG	T–/T–/T–
R	tRNA-Arg	10002/10009/10003	10070/10077/10071	69/69/69	0/0/0	F		
	ND4L	10071/10078/10072	10367/10374/10368	297/297/297	–7/–7/–7	F	ATG/ATG/ATG	TAA/TAA/TAA
	ND4	10361/10368/10362	11741/11748/11742	1381/1381/1381	0/0/0	F	ATG/ATG/ATG	T–/T–/T–
H	tRNA-His	11742/11749/11743	11810/11817/11811	69/69/69	0/0/0	F		
S	tRNA-Ser	11811/11818/11812	11878/11885/11879	68/68/68	+11/+8/+8	F		
L	tRNA-Leu	11890/11894/11888	11962/11966/11960	73/73/73	0/0/0	F		
	ND5	11963/11967/11961	13801/13805/13799	1839/1839/1839	–4/–4/–4	F	ATG/ATG/ATG	TAA/TAA/TAA
	ND6	14319/14323/14317	13798/13802/13796	522/522/522	0/0/0	R	TAC/TAC/TAC	ATC/ATC/ATC
E	tRNA-Glu	14388/14392/14386	14320/14324/14318	69/69/69	+4/+8/+5	R		
	CYTB	14393/14401/14392	15533/15541/15532	1141/1141/1141	0/0/0	F	ATG/ATG/ATG	T–/T–/T–
T	tRNA-Thr	15534/15542/15533	15606/15615/15604	73/74/72	+1/+1/0	F		
P	tRNA-Pro	15677/15687/15674	15608/15617/15605	70/71/70	0/0/0	R		
	Control region	15678/15688/15675	16519/16547/16523	842/860/849	0/0/0	F		

The overall base composition is 29.2% A, 48.9% C, 16.3% G, 25.5% T, and 45.2% GC for *E. amblyopsis*. For *E. picta*, the base composition is 27.6% A, 30.9% C, 17.7% G, 23.8% T, and 48.6% GC. For *H. latifasciata*, the overall base composition is 31% A, 36.3% C, 10.1% G, 22.6% T, and 46.4% GC.

All protein-coding genes are of identical length across the three species, with the exception of ATP8 that is reduced by a single codon in *E. picta*. Non-coding regions, on the other hand, show more variation in length. For example, seven out of 22 tRNAs differ in size by a single base pair (bp): *E. amblyopsis* is one bp longer in tRNA-Trp, and one bp shorter in tRNA-Tyr; *E. picta* has an additional bp in tRNA-Pro and tRNA-Asp; *H. latifasciata* is one bp shorter in tRNA-Val, tRNA-Met, tRNA-Asn, and tRNA-Thr. 16S rRNA and control region differ drastically among the three species, ranging between 1687 and 1695 bp for the 16S rRNA, and 842 and 860 b for the control region. Within species, the two *E. picta* specimens exhibit 58 mutational differences throughout their mitochondrial genomes; 15 of these changes are found in non-coding regions, while the translation of eight codons differs as a result of these mutations.

The most common initiation codon, ATG, is found in all the mitochondrial protein-coding genes apart from COXI, that has GTG (Val), and ND6, that has TAC (Tyr), as start codons in all species. Five genes share the common termination codon TAA across all species. In ND1, *E. amblyopsis* and *E. picta* differ in having the termination codon TAG, while in *E. picta* remains TAA. For ND6, ATC is the stop codon in every species. COXII, COXIII, ND3, ND4, and CYTB coding regions have incomplete stop codons: T or TA. These incomplete stop codons are completed as TAA by the post-transcriptional polyadenylation of the corresponding mRNAs (Ojala et al. 1981).

**Figure 1.**
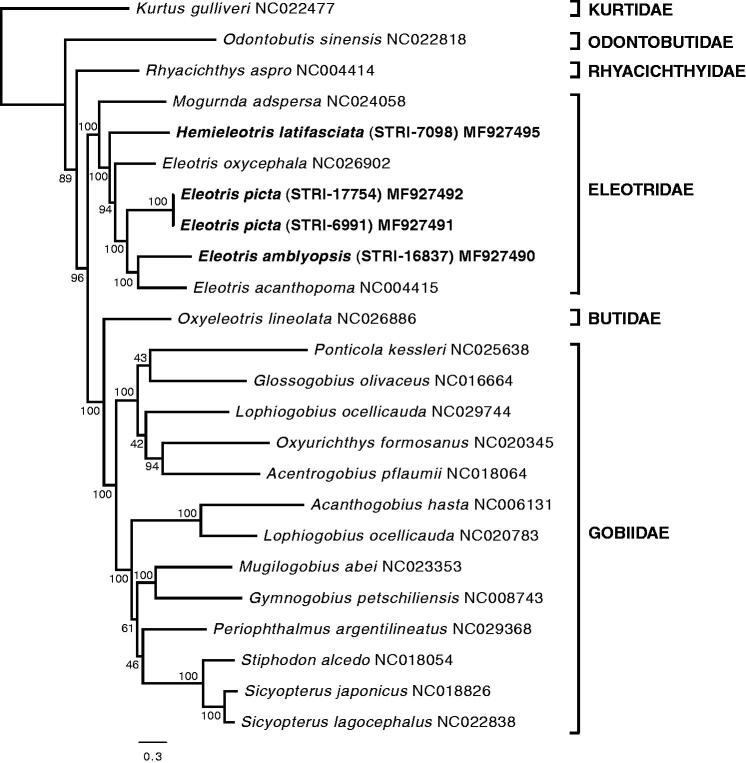
Maximum likelihood (RAxML) phylogeny using all mitochondrial protein-coding genes from a selection of Gobiiformes species from which complete mitogenomes are available (GenBank accession numbers indicated), and *Kurtus gulliveri*, which was used as outgroup. Numbers next to nodes are support values obtained after 500 bootstrap replicates. New sequences obtained in this study are highlighted in bold.

Our phylogenetic reconstruction strongly supports the monophyly of the family Eleotridae. *Hemieleotris latifasciata* is the sister group to a clade containing all the species of the genus *Eleotris*. Neotropical members of *Eleotris* do not form a monophyletic group: *E. picta*, from the Eastern Pacific, is the sister species to the clade formed by *E. amblyopsis*, distributed in the East Atlantic, and *E. acanthopoma*, which is widespread across the South West Pacific. *Eleotris oxycephala* from the West Pacific, is the sister taxon to the least inclusive clade that contains all the Neotropical members of *Eleotris* (Figure 1). We resist drawing any major biogeographic conclusion at this time, given the limited sampling, but do point out that major transoceanic relationships do exist in Gobiiformes (Chakrabarty et al. 2012).

Here, we present the first mitogenomes of three members of the Eleotridae from the Neotropics. These sequences will aid future studies into the evolutionary relationships of gobiiform fishes. This work will also aid our understanding of the diversity of Middle America freshwater fish assemblages as mitochondrial genomes of additional species and lineages become available.
